# Dietary Phytochemicals and Depressive Symptoms in Young Adults: Evidence from Undergraduate Students in Türkiye

**DOI:** 10.3390/nu17213406

**Published:** 2025-10-29

**Authors:** Yagmur Yasar Firat, Betul Cicek

**Affiliations:** Department of Nutrition and Dietetics, Faculty of Health Sciences, Erciyes University, Kayseri 38260, Türkiye; bcicek@erciyes.edu.tr

**Keywords:** depression, dietary phytochemical index, phytochemicals, undergraduate students

## Abstract

**Background/Objectives:** Depression is a prevalent mental health problem among undergraduate students, and dietary patterns may play a role in its prevention. Phytochemical-rich diets have been proposed to be potential protective factors against depression due to their antioxidant, anti-inflammatory, and neuroprotective properties. This study aimed to investigate the association between the Dietary Phytochemical Index (DPI) and depressive symptoms among undergraduate students in Türkiye. **Methods:** A descriptive, cross-sectional study was conducted among 789 undergraduate students at Erciyes University between May 2024–May 2025. Dietary data were collected using a 101-item Food Frequency Questionnaire, and the DPI was calculated as the percentage of total daily energy derived from phytochemical-rich foods. Depressive symptoms were assessed via the Burns Depression Checklist (BDC). Statistical analyses included correlation and logistic regression models adjusted for gender, income, and academic department. **Results:** Participants with higher DPI scores exerted significantly lower BDC total and sub-dimension scores, including activities and personal relationships, physical symptoms, and suicidal urges (all *p* < 0.05). The inverse association between DPI and total depression score remained significant across all adjusted models (*p* < 0.001), and a significant linear trend was observed across DPI quartiles (*p*-trend < 0.001). **Conclusions:** Higher dietary phytochemical intake was associated with lower depressive symptom levels among undergraduate students. These results suggest that phytochemical-rich dietary patterns, characterized by increased consumption of fruits, vegetables, whole grains, legumes, and nuts, may contribute to improved psychological well-being. Promoting the intake of phytochemical-dense foods could be a practical nutritional strategy for supporting mental health in young adults.

## 1. Introduction

Depression is a prevalent psychological condition, often defined by a persistent poor mood and reduced interest, exhibiting a high incidence rate. In recent years, the prevalence of depression has increased annually, impacting over 280 million individuals globally [1]. Currently, it affects individuals of all ages across all nations, harming not only patients but also their families and communities [2]. Undergraduate students deal with stress from their daily lives as well as from the demands of their coursework and the extra difficulties of campus life. They are considered a vulnerable group since they are in a transitional developmental stage, striving to adapt to a new environment while simultaneously coping with academic pressures [3]. Research among Turkish undergraduate students demonstrated the prevalence of depression to range between 32.8% and 73.6% [4,5,6].

Several dietary approaches and eating patterns have been examined for their possible role in preventing or alleviating depressive symptoms [7]. Findings from various studies indicated that greater consumption of healthy foods, such as vegetables and fruits, was linked to a reduced risk of depression, and greater consumption of unhealthy items, including soda, French fries, fast food, sweetened fruit drinks, bakery products (cakes, cookies, and pies), ice cream, and frozen desserts, was inversely associated with psychological distress [8,9,10]. Vegetables, fruits, whole grains, nuts, and legumes are rich in phytochemicals, such as phenolic acids, carotenoids, terpenoids, organosulfur compounds, and phytosterols, that function as antioxidants [11]. Evidence from experimental research suggests that phytochemicals possess antidepressant activity [12,13].

Globally, depression is one of the main causes of disability, and it especially affects young adults who are subjected to social, economic, and academic pressures. Alongside psychosocial impacts, growing data indicate that nutritional factors significantly influence the etiology and therapy of depressive disorders [14]. Diets rich in plant-based foods and bioactive chemicals, including polyphenols, flavonoids, and carotenoids, may alleviate oxidative stress, inflammation, and neurotransmitter abnormalities, all of which are associated with the pathophysiology of depression [15]. Consequently, investigating the density of dietary phytochemicals in relation to mental health outcomes among university students is becoming important from both clinical and public health approaches [11]. Considering that undergraduate students are a potential risk group for depression, the present study aims to investigate the effect of dietary phytochemical content on depression within this population.

## 2. Methods

### 2.1. Study Design and Sampling Method

This descriptive, cross-sectional study was conducted between May 2024 and May 2025. According to the 2023 data from the Council of Higher Education, Erciyes University has 38088 undergraduate students. The required sample size was calculated using the Epi Info™ program (Division of Health Informatics & Surveillance, CDC, Atlanta, GA, USA). Previous studies reported that the prevalence of depression among Turkish undergraduate students ranged from 32.8% to 73.6% [4,5,6]. Assuming a mean prevalence of 50%, with a significance level of α = 0.05, a design effect of 4 (to account for allocation into DPI quartiles), and 80% statistical power, the minimum sample size was estimated as 656. Considering a 20% potential non-response rate, a total of 789 students were included. Undergraduate students were recruited from all faculties of Erciyes University. Official permission letters were sent to the deans of each faculty, and, after approval, the researchers visited classrooms to administer the questionnaire in person. Within each faculty, students were selected randomly, and the number of questionnaires distributed was determined proportionally to the total number of students enrolled in each faculty. Data collection continued until the target sample size, as determined by the power analysis, was achieved. The final sample size (*n* = 789) accounted for potential data losses due to incomplete responses. Exclusion criteria were the presence of chronic diseases (e.g., diabetes mellitus, insulin resistance, hypertension, ischemic heart disease, thyroid disorders, chronic kidney or liver disease), acute illnesses (e.g., infection, flu, cold), adherence to special diets for medical reasons or weight loss, pregnancy, and the use of any dietary supplements. In addition, participants with a prior diagnosis of any psychiatric or neurological disorder or those receiving psychotropic medication were excluded.

### 2.2. Ethical Considerations

Approval for the study was obtained from the Social Sciences Ethics Committee of Erciyes University (Approval number: 185, Date: 30 April 2024). The study was carried out in accordance with the Declaration of Helsinki, and written informed consent was obtained from all participants after providing information about the purpose of the study. During data collection, all participants were informed that the questionnaire included items related to depressive and suicidal thoughts. Participation was voluntary and anonymous. In cases where participants indicated severe depressive symptoms or suicidal urges, the research team immediately provided information about available on-campus psychological counseling and mental health support services.

### 2.3. Data Collection Tools

Data were collected using a face-to-face interview method from voluntary participants using a structured questionnaire consisting of three sections:

1. Sociodemographic and Nutritional Behavior Information: Questions on personal characteristics, nutritional habits, and dietary supplement use.
2. Burns Depression Checklist (BDC): Developed by David Burns, the BDC is a reliable and widely used tool for assessing depressive symptoms. The Turkish validity and reliability study was conducted by Tuncer and Dikmen [16]. The scale includes 25 items covering emotional and cognitive symptoms (items 1–10), activities and personal relationships (items 11–17), physical symptoms (items 18–22), and suicidal ideation (items 23–25). Responses are scored on a 5-point Likert scale from 0 (“not at all”) to 4 (“extremely”), based on experiences during the past week. Total scores range from 0 to 100, with the following classifications: 0–5 = no depression, 6–10 = normal but unhappy, 11–25 = mild depression, 26–50 = moderate depression, 51–75 = severe depression, and 76–100 = extreme depression [16].
3. Food Frequency Questionnaire (FFQ): A semi-quantitative FFQ including 101 items was used to evaluate dietary intake and calculate the Dietary Phytochemical Index (DPI).

#### Dietary Phytochemical Index (DPI)

The DPI was calculated based on the method proposed by McCarty [17]:

DPI = [(daily energy intake from phytochemical-rich foods (kJ)/total daily energy intake (kJ)) × 100].

Phytochemical-rich foods included fruits, vegetables, legumes, whole grains, nuts, soy products, seeds, and olive oil. Natural fruit and vegetable juices and tomato sauces were also classified within fruit and vegetable groups, due to their high phytochemical content (e.g., polyphenols, carotenoids, isoflavones, and lignans). A detailed list of all FFQ items and their classification is provided in Supplementary Table S1.

Consumption frequencies were converted into daily equivalents using standard coefficients: “per meal” = 3, “every day” = 1, “5–6 times per week” = 0.7855, “3–4 times per week” = 0.498, “1–2 times per week” = 0.2145, “once every 15 days” = 0.067, and “once per month” = 0.033. These values were then analyzed using the BEBİS program (Nutrition Information System, Computer-Aided Nutrition Program) to calculate daily energy, macronutrient, and micronutrient intake [18].

### 2.4. Statistical Analysis

Statistical analyses were performed using SPSS for Windows, version 22.0. A *p*-value of <0.05 was considered as statistically significant. Normality of scale scores was assessed using the Kolmogorov–Smirnov test. Descriptive statistics were expressed as the mean ± standard deviation for normally distributed variables and median (minimum–maximum) for non-normally distributed variables. Parametric tests were applied for normally distributed variables, while non-parametric tests were used otherwise. Differences among DPI quartiles were evaluated using the Kruskal–Wallis test, followed by Dunn’s post hoc test with Bonferroni correction. Relationships between categorical variables were analyzed using the chi-square test, and correlations were examined with correlation analyses. Correlations between the DPI and psychological variables were examined using Spearman’s rank correlation test. Multiple linear regression analyses were performed to examine the association between depressive symptom scores and DPI quartiles after adjusting for potential confounders. *p*-for-trend values were calculated by entering the median DPI value of each quartile as a continuous variable in the model.

Use of AI-assisted technologies: During the manuscript preparation, QuillBot (https://quillbot.com/, accessed on 8 October 2025) was used for paraphrasing assistance, and DeepL Translator (https://www.deepl.com/en/translator, accessed on 8 October 2025) was used for the English translation. The authors reviewed and edited all content as needed and take full responsibility for the integrity and accuracy of the final manuscript.

## 3. Results

Of the 789 students included, 74% were females (*n* = 584) and 26% were males (*n* = 205). The mean age was 22.82 ± 4.22 years, and the mean DPI score was 27.23 ± 10.63.

As shown in Table 1, the students’ total and sub-dimension scores on the BDC differed significantly across DPI quartiles. Participants in the lowest DPI quartile (Q1) had the highest mean scores in the sub-dimensions of thoughts and feelings, activities and personal relationships, physical symptoms, and suicidal urges, as well as the highest total depression scores. In contrast, these scores decreased progressively across higher DPI quartiles. Significant reductions were observed in thoughts and feelings (*p* = 0.021), activities and personal relationships (*p* < 0.001), physical symptoms (*p* = 0.003), suicidal urges (*p* < 0.001), and total score (*p* < 0.001). These findings indicate that a diet rich in phytochemicals is associated with lower levels of depressive symptoms.

The distribution of participants’ emotional states according to DPI quartiles is presented in Table 2. Although emotional states differed across DPI quartiles, the difference was not significant (*p* = 0.102). Nevertheless, the proportion of participants without depression or with mild depression was higher in the upper DPI quartiles (Q3–Q4), whereas the prevalence of moderate and severe depression was greater in the lower quartiles (Q1). This trend suggests that higher DPI levels may be associated with more favorable emotional states.

The correlations between DPI scores and the total and sub-dimension scores of the BDC are shown in Table 3. Statistically significant negative correlations were detected between DPI and activities and personal relationships (*r* = −0.082; *p* = 0.021), physical symptoms (*r* = −0.071; *p* = 0.047), suicidal urges (*r* = −0.195; *p* < 0.001), and total score (*r* = −0.096; *p* = 0.007). Although the correlation between DPI and thoughts and feelings was not significant (*r* = −0.069; *p* = 0.054), a similar negative tendency was observed. These results support that higher DPI levels are associated with fewer depressive symptoms.

When the relationship between participants’ DPI scores and their BDC total and sub-dimension scores is examined, it is seen that DPI was significantly and negatively associated with activities and personal relationships (β = −0.146; 95% CI: −0.269 to −0.022; *p* = 0.021), physical symptoms (β = −0.185; 95% CI: −0.368 to −0.003; *p* = 0.047), suicidal urges (β = −1.287; 95% CI: −1.741 to −0.834; *p* < 0.001), and total score (β = −0.059; 95% CI: −0.102 to −0.016; *p* = 0.007) (Table 4).

When participants were classified according to DPI quartiles (Table 5), a clear inverse relationship between DPI and total depression scores was observed. Compared with the lowest DPI quartile (Q1), total depression scores were significantly lower in Q2, Q3, and Q4 (β = −6.20; −6.27; −5.38; all *p* < 0.01). This relationship remained significant after adjustment for gender (Model 1), income level (Model 2), and attending a health-related department (Model 3) (*p* < 0.001; *p* = 0.002; *p* = 0.002, respectively). In the fully adjusted model (Model 4), this significant negative association persisted (Q2 = −6.44; Q3 = −6.35; Q4 = −5.79; all *p* < 0.001). Moreover, a significant linear trend was observed across DPI quartiles (*p*-trend < 0.001). These results suggest that a diet rich in phytochemicals may contribute to lower depressive symptom scores among young adults.

## 4. Discussion

The present study investigated the relationship between the DPI and depressive symptoms among undergraduate students. The results revealed that higher DPI levels were significantly associated with lower scores on the BDC and its sub-dimensions, including thoughts and feelings, activities and personal relationships, physical symptoms, and suicidal urges. These results suggest that a diet rich in phytochemical-containing foods may play a protective role against depressive symptoms in young adults.

Our current findings align with other studies suggesting that increased consumption of phytochemical-rich foods, including fruits, vegetables, legumes, and whole grains, is negatively correlated with depression and psychological distress. A study by Sangouni et al. [11], including Iranian adolescent females, found a strong negative correlation between DPI and depressive symptoms, as well as quality of life, corroborating the idea that diets high in phytochemicals may enhance mental well-being. Banta et al. [8] and Wattick et al. [9] similarly showed that individuals consuming diets rich in plant-based foods had improved mental health and a reduced incidence of depression relative to those with poor dietary quality. A large-sample study conducted in Iran with adults reported a significant inverse association between DPI and symptoms of depression and anxiety. Among normal-weight individuals, those in the highest tertile of DPI had a significantly lower risk of depression and anxiety than those in the lowest tertile. However, this association was not observed in overweight and obese individuals. These findings suggest that phytochemical-rich diets may have a protective effect on mental health; however, this effect may vary depending on individual body mass index [19].

The results of the current study are consistent with growing evidence suggesting a protective role of phytochemicals, particularly polyphenols and flavonoids, against depression. Phytochemicals, including polyphenols, flavonoids, carotenoids, and organosulfur compounds, possess potent antioxidant and anti-inflammatory properties that may counteract the chronic inflammation and oxidative stress implicated in the pathophysiology of depression [10,20]. Polyphenols exert neuroprotective and neuromodulatory effects by regulating cellular signaling, gene expression, and neurotransmitter systems [21,22,23]. Some polyphenols, known as phytoestrogens, may also influence mood through their interaction with estrogen receptors and modulation of monoaminergic enzymes such as tryptophan hydroxylase and monoamine oxidase [24,25]. Experimental and observational evidence further supports these mechanisms. Melo et al. [13] demonstrated the antidepressant-like effects of carvacrol via dopaminergic modulation, while resveratrol was shown to alleviate depressive behaviors through monoaminergic regulation and suppression of neuroinflammation [12]. Consistent with these findings, Chang et al. [20] observed inverse associations between the intake of specific flavonoid subclasses (flavonols, flavones, and flavanones) and the incidence of depression, reinforcing the potential role of flavonoid-rich diets in promoting mental well-being. Collectively, our data indicate that diets with elevated DPI values, reflecting a higher intake of phytochemical-dense foods, may significantly contribute to enhancing psychological well-being and reducing depressive symptoms.

Behavioral and psychological factors, in addition to these biological mechanisms, may clarify the correlation between dietary phytochemical consumption and depression. Individuals with higher DPI values generally consume a more nutritious diet, characterized by increased intake of fruits, vegetables, and whole grains, alongside a reduced consumption of ultra-processed foods. This pattern indicates the presence of other healthy behaviors, such as regular eating habits, adherence to a high-quality diet, and self-regulation of food intake [26,27]. These characteristics are acknowledged to improve emotional stability and reduce psychological distress. Our findings suggest that even small improvements in diet quality and composition might beneficially affect mood and emotional well-being.

Recent data indicate that psychobiological systems, such as the gut–brain axis, serve as an additional link between nutrition and mood. Enhanced intake of dietary fiber and polyphenols may positively influence gut microbiota diversity, subsequently increasing the production of neuroactive compounds, such as serotonin precursors and, short-chain fatty acids, which regulate mood and emotional functioning [28,29]. Thus, the protective influence of phytochemical-rich diets may involve both direct neurochemical and indirect psychobiological interactions.

In our study, the total score of BDC and its sub-dimensions (especially activity–interpersonal relations, physical symptoms and suicidal urges) decreased significantly with the increase in DPI; trend analysis using *p*-for-trend values also supported a linear decrease in depression scores across DPI quartiles. The significant/negative relationship of the DPI with the sub-dimensions, particularly the suicidal urges score, suggests a potential protective role even at the extremes of the severity spectrum; however, this finding is scale-based rather than clinically diagnostic and should be interpreted with caution.

This study has several strengths that enhance the reliability of its findings. First, the sample size was determined using the Epi Info program, considering design effects, and the use of validated measurement tools (the Burns Depression Checklist and the detailed Food Frequency Questionnaire) supports the robustness of the results. Furthermore, the DPI, calculated based on a well-established method for assessing dietary phytochemical density, was a significant indicator of dietary quality. The consistent inverse relationship observed between DPI quartiles and significant *p*-trend values provides strong evidence supporting a linear trend between phytochemical-rich dietary patterns and depressive symptoms.

However, the study also has some limitations. First, the cross-sectional design precludes establishing a cause-and-effect relationship and carries the risk of reverse causality due to the possibility that depressed individuals may have altered their food preferences. The fact that participants’ dietary data were self-reported may have led to recall bias and measurement error. The DPI, being an energy-based index, may not fully reflect the impact of low-energy but phytochemical-dense foods (e.g., spices, herbal teas). Although adjustments were made for many confounding variables, the influence of unmeasured factors such as physical activity, sleep quality, stress levels, or medication use cannot be excluded. In addition, the study was conducted at a single university with a predominantly female sample, which limits the generalizability of the results. Data collection, however, was carried out outside of examination periods, as the researchers were also assigned as exam proctors during those times. Therefore, the potential influence of exam-related stress on mood and dietary behaviors was minimized.

Future research must be designed as prospective cohort and randomized-controlled trials to confirm a causal relationship. Furthermore, measuring biochemical markers related to oxidative stress, inflammation, and monoamine metabolism will contribute to a better understanding of the biological mechanisms underlying this relationship. Investigating the interactions of phytochemicals with other dietary components, such as omega-3 fatty acids or dietary fiber, can provide a more comprehensive view of the diet-depression relationship. Furthermore, intervention studies aimed at increasing the consumption of phytochemical-rich foods among undergraduate students could provide valuable information for nutrition-based mental health promotion strategies.

## 5. Conclusions

In conclusion, this study demonstrated a significant inverse association between DPI and depressive symptoms among undergraduate students. Participants with higher DPI scores, reflecting greater consumption of phytochemical-rich foods such as fruits, vegetables, whole grains, legumes, and nuts, exhibited lower levels of depression and its sub-dimensions. These findings suggest that diets abundant in plant-based and minimally processed foods may play a beneficial role in mental well-being, potentially through antioxidant, anti-inflammatory, and neuroprotective mechanisms associated with phytochemicals.

Although the cross-sectional design of the study precludes causal inference, the significant linear trend observed across DPI quartiles suggests a potential dose-response association between phytochemical-rich dietary patterns and depressive symptoms. Encouraging the consumption of phytochemical-dense foods within the university population may represent a promising and practical approach to promote mental health and reduce the burden of depression among young adults. Future longitudinal and interventional studies are warranted to confirm these associations and to elucidate the underlying biological mechanisms.

## Figures and Tables

**Table 1:** BDC sub-dimensions and total score of participants according to DPI quartiles.

BDC	Q1 (*n* = 197)	Q2 (*n* = 197)	Q3 (*n* = 198)	Q4 (*n* = 197)	*p*
Thoughts and feelings	13.00 ^a^ (0.00–36.00)	10.00 ^b^ (0.00–40.00)	10.00 (0.00–35.00)	10.00 (0.00–40.00)	0.021
Activities and personal relationships	10.00 ^a^ (0.00–26.00)	7.00 ^b^ (0.00–28.00)	7.00 ^b^ (0.00–28.00)	8.00 ^b^ (0.00–28.00)	*p* < 0.001
Physical symptoms	7.00 ^a^ (0.00–20.00)	6.00 ^b^ (0.00–20.00)	5.00 ^b^ (0.00–28.00)	6.00 (0.00–16.00)	0.003
Suicidal urges	0.00 ^a^ (0.00–8.00)	0.00 ^a^ (0.00–8.00)	0.00 ^b^ (0.00–7.00)	0.00 ^b^ (0.00–8.00)	*p* < 0.001
Total score	30.00 ^a^ (0.00–84.00)	24.00 ^b^ (0.00–96.00)	24.00 ^b^ (0.00–76.00)	24.00 ^b^ (0.00–80.00)	*p* < 0.001

Note: BDC, Burn’s Depression Checklist; DPI, Dietary Phytochemical Index. Different superscript letters (^a^, ^b^) indicate significant differences between groups according to Dunn’s post hoc test with Bonferroni correction (*p* < 0.05).

**Table 2:** Distribution of participants’ emotional states according to DPI quartiles.

Emotional State	Q1 (*n* = 197)		Q2 (*n* = 197)		Q3 (*n* = 198)		Q4 (*n* = 197)		Total (*n* = 789)		*p*
	n	%	n	%	n	%	n	%	n	%	
No depression	9	12.3	25	34.2	25	34.2	14	19.2	73	100	0.102
Normal but unhappy	14	23.3	17	28.3	15	25.0	14	23.3	60	100	
Mild depression	60	22.2	65	24.1	69	25.6	76	28.1	270	100	
Moderate depression	84	27.6	75	24.7	68	22.4	77	25.3	304	100	
Severe depression	28	37.3	14	18.7	19	25.3	14	18.7	75	100	
Extreme depression	2	28.6	1	14.3	2	28.6	2	28.6	7	100	

Note: DPI, Dietary Phytochemical Index.

**Table 3:** Correlation between participants’ DPI scores and BDC’s sub-dimensions and total scores.

BDC	DPI	
	*r*	*p*
Thoughts and feelings	−0.069	0.054
Activities and personal relationships	−0.082	0.021
Physical symptoms	−0.071	0.047
Suicidal urges	−0.195	*p* < 0.001
Total score	−0.096	0.007

Note: BDC, Burn’s Depression Checklist; DPI, Dietary Phytochemical Index. Spearman’s rank correlation test was used.

**Table 4:** The relationship between the participants’ total and sub-dimension scores of the BDC and DPI scores.

BDC	DPI			
	β	95% CI		*p*
		Lower	Upper	
Thoughts and feelings	−0.092	−0.185	0.002	0.054
Activities and personal relationships	−0.146	−0.269	−0.022	0.021
Physical symptoms	−0.185	−0.368	−0.003	0.047
Suicidal urges	−1.287	−1.741	−0.834	*p* < 0.001
Total score	−0.059	−0.102	−0.016	0.007

Note: BDC, Burn’s Depression Checklist; DPI, Dietary Phytochemical Index. DPI ranged from 1.8 to 63.38. Quartile cut points and mean ± SD values for the DPI were as follows: Q1 = 1.8–20.36 (15.10 ± 4.25), Q2 = 20.37–26.07 (23.19 ± 1.69), Q3 = 26.08–33.28 (29.22 ± 2.02), and Q4 = 33.29–63.38 (41.39 ± 7.71).

**Table 5:** Association between depression and quartiles of DPI according to multiple linear regression analysis.

	Quartiles of DPI				*p*-Trend
	Q1	Q2	Q3	Q4	
Crude	1.00	−6.44 (−9.79, −3.08)	−6.27 (−9.63, −2.90)	−5.38 (−8.74, −2.01)	0.003
Model 1	1.00	−6.44 (−9.79, −3.08)	−6.45 (−9.80, −3.10)	−5.98 (−9.35, −2.60)	*p* < 0.001
Model 2	1.00	−6.18 (−9.54−2.81)	−6.19 (−9.55, −2.83)	−5.22 (−8.60, −1.85)	0.002
Model 3	1.00	−6.19 (−9.56, −2.83)	−6.27 (−9.64, −2.91)	−5.39 (−8.76, −2.02)	0.002
Model 4	1.00	−6.41 (−9.77, −3.06)	−6.35 (−9.70, −3.00)	−5.79 (−9.17, −2.40)	*p* < 0.001

Note: DPI, Dietary Phytochemical Index; Model 1 = adjusted for gender; Model 2 = adjusted for income level; Model 3 = adjusted for health-related department; Model 4 = adjusted for Model 1, Model 2 and Model 3. Data are presented as β coefficients with 95% confidence intervals (CI). *p* for-trend values were calculated by treating DPI quartiles as an ordinal variable.

## Data Availability

The datasets used and/or analyzed during the current study are available from the corresponding author on reasonable (ethical reasons) request.

## Supplementary Materials

The following supporting information can be downloaded at: https://www.mdpi.com/article/10.3390/nu17213406/s1, Table S1. Classification of FFQ items according to the McCarty method.

## Abbreviations

The following abbreviations are used in this manuscript:

DPI	Dietary Phytochemical Index
BDC	Burns Depression Checklist
FFQ	Food Frequency Questionnaire
CI	Confidence Interval
