# Supplementary material for: Dietary Phytochemicals and Depressive Symptoms in Young Adults: Evidence from Undergraduate Students in Türkiye
Source: Nutrients. 2025 Oct 29;17(21):3406. doi: 10.3390/nu17213406 (PMC12608252; doi:10.3390/nu17213406)
Supplement: Supplementary file 1 [file nutrients-17-03406-s001.zip › nutrients-3947533-supplementary.pdf]

Table S1. Classification of FFQ items according to the McCarty method.

Foods	Classified as phytochemical-rich?
**Meat, eggs, legumes**	
Beef (Full-fat)	No
Beef (Semi-fat)	No
Beef (Lean)	No
Lamb	No
Chicken (Breast)	No
Chicken (Drumstick)	No
Chicken (Skin-on)	No
Chicken (Thigh/Drumstick)	No
Chicken (Skin-on wings)	No
Fish	No
Eggs	No
Turkish sausage	No
Salami	No
Sausage	No
Pastrami	No
Liver (Beef)	No
Liver (Lamb)	No
Chickpeas	Yes
Dried beans	Yes
Lentils (Green/red)	Yes
Sunflower seeds	Yes
Pumpkin seeds	Yes
Hazelnuts	Yes
Peanuts	Yes
Pistachios	Yes
Cashews	Yes
Walnuts	Yes
Almonds	Yes
**Milk and dairy products**	
Whole milk	No
Low-fat milk	No
Skim milk	No
Whole milk yogurt	No
Low-fat yogurt	No
Skim yogurt	No
Whole-milk white cheese	No
Low-fat white cheese	No
Cheddar Cheese	No
Çökelek (cottage cheese)	No
Goat cheese	No
Cream cheese	No
Cottage cheese	No
**Fresh vegetables and fruits**	
Group A vegetables	Yes
Group B vegetables	Yes
Potatoes	No
Citrus fruits	Yes
Other fruits	Yes

**Bread and grains**	
White bread	No
Whole wheat bread	Yes
Whole grain breads	Yes
Yufka	No
Bazlama	No
Rice	No
Bulgur	Yes
Pasta	No
Tarhana	Yes
Biscuits/crackers	No
Simit	No
Whole grain breakfast cereals	Yes
**Oil and sugar**	
Sugar and sugar substitutes	No
Honey	No
Jam	No
Molasses	No
Chocolate	No
Turkish delight	No
Pastries	No
Desserts made with milk	No
Olive oil	Yes
Hazelnut oil	No
Sunflower oil	No
Corn oil	No
Soybean oil	Yes
Canola oil	No
Butter	No
Hard margarine	No
Soft margarine	No
Tallow/lard	No
Chips	No
Mayonnaise	No
Olives	Yes
Beverages	No
Ready-made fruit juice	No
Fresh fruit Juice	Yes
Carbonated drinks	No
Mineral water	No
Tea	No
Herbal teas	No
Instant coffee (Nescafe)	No
Turkish coffee	No
Filter coffee	No
Beer	Yes
Wine	Yes
Raki	No
Whiskey, gin	No

Others	
Ready-made soup	No
Pide (with meat) / lahmacun	No
Pide (with cheese)	No
Pizza	No
Pizza (with vegetables)	No
Döner	No
Kebab	No
Hamburger	No
Fried chicken pieces	No

Group A vegetables: Broccoli, cabbage, cauliflower, zucchini, spinach, okra, green beans, eggplant, purslane, Swiss chard

Group B vegetables: Carrots, artichokes, pumpkin, peas, broad beans, leeks, fava beans, Jerusalem artichokes, Brussels sprouts, red peppers, celery
